# Long-term follow-up of therapeutic efficacy of everolimus-eluting bioresorbable vascular scaffold in comparison to everolimus-eluting stent in treatment of chronic total occlusion guided by intracoronary imaging

**DOI:** 10.1186/s43044-020-00104-x

**Published:** 2020-10-21

**Authors:** Mohammad Abdallah Eltahlawi, Abdel-Aziz Fouad Abdel-Aziz, Abdel-Salam Sherif, Khalid Abdel-Azeem Shokry, Islam Elsayed Shehata

**Affiliations:** 1 Department of Cardiology, Faculty of Medicine, Zagazig University, Zagazig, 44519 Egypt; 2 Department of Cardiology, Kobry AlKobah Military Hospital, Military Medical Academy, Cairo, Egypt; 3 College of Medicine, Department of Cardiology, Military Medical Academy, Cairo, Egypt

**Keywords:** Bioresorbable vascular stents, Chronic total occlusion, Coronary artery disease, Drug-eluting stent, Intracoronary imaging, Multi-slice CT coronary angiography

## Abstract

**Background:**

We hypothesized that 1st generation everolimus-eluting bioresorbable vascular scaffold (BVS) stent associated with less complication and less restenosis rate than everolimus-eluting stent (EES) in chronic total occlusion (CTO) recanalization guided by intracoronary imaging. Therefore, we aimed to assess the safety and performance of BVS stent in CTO revascularization in comparison to EES guided by intracoronary imaging.

Our prospective comparative cross-sectional study was conducted on 60 CTO patients divided into two groups according to type of stent revascularization: group I (EES group): 40 (66.7%) patients and group II (BVS group): 20 (33.3%) patients. All patients were subjected to history taking, electrocardiogram (ECG), echocardiography, laboratory investigation, stress thallium study to assess viability before revascularization. Revascularization of viable CTO lesion guided by intracoronary imaging using optical coherence tomography (OCT). Then, long-term follow-up over 1 year clinically and by multi-slice CT coronary angiography (MSCT). Our clinical and angiographic endpoints were to detect any clinical or angiographic complications during the follow-up period.

**Results:**

At 6 months angiographic follow-up, BVS group had not inferior angiographic parameters but without statistically significant difference (*p* = 0.566). At 12 months follow-up, there was no difference at end points between the two groups (*p* = 0.476).

No differences were found at angiographic or clinical follow-up between BVS and EES.

**Conclusion:**

This study shows that 1st generation everolimus-eluting BVS is non-inferior to EES for CTO revascularization. Further studies are needed to clearly state which new smaller footprint BVS, faster reabsorption, magnesium-based less thrombogenicity, and advanced mechanical properties is under development. We cannot dismiss the efficacy and safety of new BVS technology.

**Trial registration:**

ZU-IRB#2498/3-12-2016 Registered 3 December 2016, email: IRB_123@medicine.zu.edu.eg

## Background

Coronary artery disease (CAD) has been the main cause of death in the world [1, 2]. The incidence of CAD is increasing yearly more in younger patients [3, 4]. Coronary angiography remains the clinical gold standard for diagnosis of coronary artery disease [5]. Currently drug-eluting stents (DES) used for revascularization of CAD with significant stenosis (> 70%) [6]. Drug carriers of DES are mainly polymer coatings, which are developed to carry enough anti-proliferation drug dosage and can perfectly control the degradation, penetration and release of everolimus or other drugs. The newly invented bioabsorbable vascular stent (BVS) have a number of benefits [7, 8]. First, BVS opens the occlusion of coronary artery. Second, after its absorption, BVS can restore endothelial function and normal vasomotion. Third, avoid a “full metal jacket,” precluding future coronary artery bypass graft (CABG). Previous studies have been conducted to compare the efficacy of BVS with DES but the outcomes were inconsistent and remain to be identified [9–12].

We hypothesized that BVS associated with less complication and less restenosis rate than EES in CTO recanalization guided by intracoronary imaging. Therefore, we aimed to assess the safety and performance of BVS stent in CTO revascularization in comparison to EES guided by intracoronary imaging.

## Methods

### Study site

Our study was conducted at the cardiology department of our hospitals.

### Time frame

Patients were recruited for 24 months from January 2017 to January 2019.

### Study population

The study group comprised 60 patients with true chronic total occlusion (CTO) diagnosed by coronary angiography whom scheduled for revascularization of CTO lesion in our hospital who fulfilled the inclusion criteria and followed up over 24 months.

### Study design

The present prospective comparative cross-sectional study included 60 CTO patients divided into two groups according to type of stent revascularization: group I (EES group): 40 (66.7%) patients and group II (BVS group): 20 (33.3%) patients.

### Sample size estimation

Assuming a frequency of 2.3% of CTO in controls and 37.4% in cases, with 80% power and 95% CI, the estimated sample was 60 patients (OPEN-EPI-Info version 6).

### Inclusion criteria

Our study included 60 true or probable CTO patients in one or more of the coronary arteries with persistence of severe symptoms (e.g., typical ischemic chest pain, Angina equivalent) despite maximum medical therapy (e.g., nitrate, anti-platelets, beta-blockers, angiotensin converting enzyme inhibitors, and statins) with evidence of viable myocardium confirmed by stress myocardial thallium study in the territory of CTO vessels.

### Exclusion criteria

Patient refusal, extremely calcified tortuous lesions resistant or non-dilatable after trials of plaque modification by cutting balloon or rotational atherectomy and confirmed by OCT evaluation, true bifurcated lesions with side branch > 2.5mm, reference vessel diameter < 2.5 mm or > 4.5 mm (out of the BVS measures), and complications treated with metallic stents.

Also we excluded patients with moderate-to-severe valvular heart disease, prosthetic heart valve, bundle branches block (LBBB or RBBB), atrial fibrillation (AF), paced rhythm, an atrioventricular block, restrictive, hypertrophic, or dilated cardiomyopathies, congenital heart disease, coronary artery ectasia, previous history of myocardial infarction, uncontrolled hypertension, hyperthyroidism, hypothyroidism, malignancy, or pulmonary, hepatic, renal, or hematological disorders.

### Tools and instruments

Resting ECG, laboratory for analysis of cardiac troponin I, fasting blood sugar, lipid profile [total cholesterol, triglycerides (TG), HDL and LDL], serum Creatinine, Creatinine clearance, C-Reactive Protein (CRP), transthoracic echocardiography (TTE), cardiac nuclear stress thallium study, coronary angiography, percutaneous coronary intervention (PCI) and optical coherence tomography (OCT), and multi-slice CT coronary angiography (MSCT).

### Study methodology

**Fig. 1:**
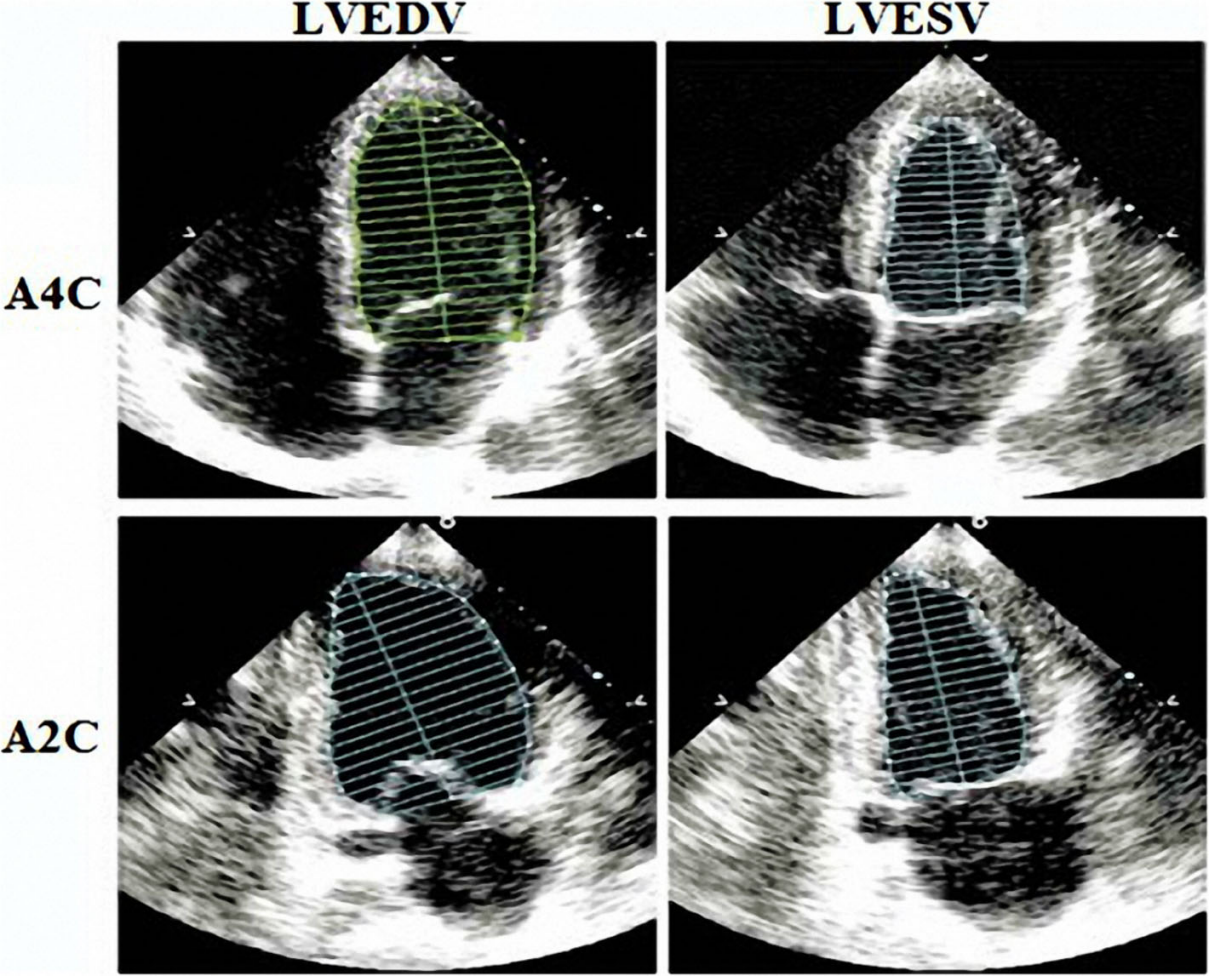
Method of calculation of biplane Simpson method. LVEDV: Left ventricular end-diastolic volume, LVESV: Left ventricular end-systolic volume, A4C: Apical 4-chamber view, A2C: Apical 2-chamber view

- All patients were subjected to the following:
  - Full history was taken with special emphasis on history of risk factors for ischemic heart disease (IHD) and family history of premature IHD, including the following:
    i. Hypertension was defined as the level of office systolic BP values are ≥ 140 mmHg and/or diastolic BP values are ≥ 90 mmHg which is equivalent to a 24-h ambulatory blood pressure (ABPM) average of ≥ 130/80 mmHg or an home blood pressure monitoring (HBPM) average of ≥ 135/85 mmHg in younger, middle-aged, and older people [13].
    ii. Diabetes mellitus was diagnosed based on presence of two criteria from the following:
      Fasting blood sugar (FBS) ≥ 126 mg/dL (7.0 mmol/L)
      Two-hour post-prandial blood sugar (2 h-PPBS) ≥ 200 mg/dL (11.1 mmol/L) during oral glucose tolerance test (OGTT) (75 g)
      HbA_1_C ≥ 6.5% (48 mmol/mol)
      Random blood sugar ≥ 200 mg/dL (11.1 mmol/L) [14].
    iii. Dyslipidemia defined in CTO (very high risk patients) if LDL-C more than 55 mg/dL (1.4 mmol/L) [15]**.**
    iv. Current smoker was defined according to the National Health Interview Survey (NHIS) as a person who reports currently smoking tobacco every day (i.e., daily smoker) or on some days (nondaily smoker) [16].
    v. Family history of CAD was defined as family history of early coronary artery disease in the first-degree relatives, male < 55 years and females < 65 years [17].
  - Thorough physical examination including pulse, heart rate, blood pressure (systolic and diastolic), neck veins, edema of lower limbs, abdominal examination, chest examinations, and cardiac examination including inspection, palpation, and auscultation.
  - A resting baseline 12-lead ECG was carried out on admission, post-PCI and at regular follow-up periods (1st, 6th, and 12th months) at a paper speed of 25 mm/s and amplification of 10 mm/mv. ECG was analyzed for: ST segment deviation, *Q* wave at number of leads with interpreted to site of old MI. Comparison with a previous ECG, when available, was valuable especially in patients with other cardiac disorders as LV hypertrophy or previous MI.
  - Transthoracic echocardiography (Fig. 1) was performed using a Vivid 9 system (GE Healthcare, Little Chalfont, UK) apparatus on admission for assessment the heart including LV ejection fraction, wall motion score index, left ventricular end-systolic volume (ESV), end-diastolic volume (EDV), and LV ejection fraction (EF) were measured from apical two and four chamber views using modified Simpson’s biplane method and the mean of the two readings was then taken. End-systole was defined as the frame with the smallest cavity area and end diastole as the frame with the largest LV cavity area. The EF was then calculated using the following formula for each view: EF (%) = [(EDV − ESV)/EDV] × 100 [18]**.**
  - Laboratory investigations including cardiac biomarkers: blood samples for troponin were collected, on presentation. Troponin I was considered positive if it exceeds the 99th percentile of normal reference (above 0.01 ng/mL), fasting blood sugar on admission, lipid profile: fasting 12 h including total cholesterol, triglycerides (TG), HDL and LDL, serum creatinine and creatinine clearance, and CBC.
  - Myocardial perfusion index (MPI): stress myocardial Thallium study done to all cases of CTO to detect myocardial viability before revascularization.
  - Coronary angiography was performed using the Judkins method. Stents were implanted using a routine method. Procedure success indicated residual stenosis < 20%, TIMI flow grade III, and no acute complication (death, myocardial infarction, emergency CABG), and no major adverse cardiac events [(cardiac death, myocardial infarction, target vessel revascularization (TVR)] in hospital. Clinical follow-up was performed at 6 and 12 months.
    - The culprit coronary lesion was clearly identified by a combination of ECG and coronary angiography. By complete occlusion of target artery TIMI 0 more than 3 months.
    - Lesion is estimated by J score which calculated from 5 points described by Christopoulos G et al. (2015) combined 5 baseline clinical and angiographic CTO parameter one point is given for each of the following factor that are associated with lower probability of successful guide wire crossing: blunt stump, calcification, lesion bending > 45°, occlusion length > 20 mm and prior failed attempt to revascularize the CTO and classified into: J-CTO = 0 is easy, J-CTO = 1 is intermediate, J-CTO = 2 is difficult, and J-CTO ≥ 3 is very difficult [19].
    - Quantitative coronary angiography (QCA) was performed in the first angiography by two independent investigators who were blinded to the results.
    - All patients are loaded by dual anti-platelet therapy by Aspirin (300 mg) and Clopidogrel (600 mg once) therapy.
    - All clinical, laboratory, and coronary angiographic data were evaluated by 2 independent investigators who were not involved in the angiographic procedures.
  - Technical steps for percutaneous coronary intervention (PCI)
    1. Preprocedural
      PCI strategy was decided to each case according to revision of coronary angiography and clinical examination (contralateral injection, guiding catheter choice, antegrade, or retro-grade approach).
    2. Intraprocedural steps
      During procedure activated clotting time was maintained above 300 ms.
        1. The procedure was done most frequently by antegrade approach in 75% in both groups and by retrograde approach in 25% in both groups if failed trial by antegrade approach.
        2. Crossing the occluded segment by guiding wire then dilatation initially by small balloon (1 mm)
        3. After nitroglycerin infusion up to (600 mcg), vessel size and lesion length were determined by quantitative coronary angiography (QCA).
        4. Pre-dilatation of entire lesion by correct balloon size.
        5. Optical coherence tomography (OCT) (Fig. 3): FD-OCT (C7-XR system) using dedicated software (Medis Qlvus 3.0 with OCT quantification software) was used to analyze morphological and anatomical characteristics before stent implantation in all cases.
        6. Optimal pre-dilatation by either cutting or noncompliant balloon to decrease risk of under expansion of stent area.
        7. Stent implantation according to OCT measurement either 1st generation BVS or 3rd generation EES.
        8. BVS implantation by increasing pressure by 2 ATM every 5 s up to 12–14 ATM with overlapping by only 1–2 mm.
        9. All BVS was deployed after good preparation using rotablation for highly fibrotic lesions and assessment of well deployment by OCT study (Fig. 3).
        10. If we need post-dilatation, this would be done using shorter NC balloon at nominal pressure with maximal increase of balloon size 0.5 mm above stent size.
        11. OCT: OCT study done with visual assessment during procedure to entire segment and NC balloon post-dilatation if any residual stenosis appeared.
    3. Post-procedural
      All patients were treated by dual anti-platelet therapy after the procedure.
  - Follow up:
    *Intraprocedural: acute procedural success was achieved by TIMI flow 3 and stenosis less than 20% without any complications. Intraprocedural complications were recorded.
    *1st, 6th and 12th month: *Clinical follow* up at 1st, 6th, and 12th month done for chest pain and hospitalization and major adverse cardiovascular events (MACE). Any death from unknown cause was considered cardiac. Evidence of myocardial damage as CK > 3 times of upper limit of normal and troponin > 5 times upper limit of normal. All patients underwent multi-slice CT coronary angiography (MSCT) after 6 months and after 12 months to detect any in-stent restenosis.

### Statistical methods

The statistical analysis was conducted following the principles as specified in International Council for Harmonization (ICH) Topic E9 (ICH1998). For continuous measurements, data were presented using mean, median, standard deviation, minimum, and maximum, and for the categorical measurements, data were presented using absolute/relative frequencies and percentages. To find the association between categorical variables, a chi-squared test or Fisher exact test was used. For all statistical analyses, a *p* value of less than or equal to 0.05 was considered to indicate a significant difference.

Correlations between variables were analyzed using the Pearson correlation coefficient. All data were analyzed using SPSS software version 22 (SPSS, Inc. Chicago, IL, USA).

## Results

### Baseline clinical characteristics

#### Demographic data (Table 1)

Baseline clinical characteristics showed approximately 90% of study were male and baseline clinical characteristics were similar between 2 groups. Mean age of patients was 57 ± 6.98 in DES group and 56.15 ± 9.25 in BVS group (*p* = 0.568).

**Table 1:** Demographic data

History (risk factors)		EES group	BVS group	Test	***p*** value	Sig.
		No. = 40	No. = 20			
Age	Mean ± SD	57.38 ± 6.98	56.15 ± 9.25	0.574^b^	0.568	NS
	Range	45–75	34–80			
Sex	Male	37 (92.5 %)	19 (95%)	0.463^b^	0.457	NS
	Female	5 (12.5 %)	1 (5%)			
DM	Negative	19 (47.5%)	8 (40.0%)	0.881^a^	0.459	NS
	Positive	21 (52.5)	12 (60.0%)			
Hyperlipidemia	Negative	5 (12.5%)	0 (0.0%)	2.727^a^	0.099	NS
	Positive	35 (87.5%)	20 (100.0%)			
HTN	Negative	17 (42.5%)	6 (30.0%)	0.881^a^	0.348	NS
	Positive	23 (57.5%)	14 (70.0%)			
History of MI	Negative	24 (60.0%)	14 (70.0%)	0.574^a^	0.449	NS
	Positive	16 (40.0%)	6 (30.0%)			
Family history of premature CAD	Negative	31 (77.5%)	17 (85.0%)	0.469^a^	0.494	NS
	Positive	9 (22.5%)	3 (15.0%)			
Previous PCI, CABG	Negative	27 (67.5%)	17 (85.0%)	2.088^a^	0.148	NS
	Positive	13 (32.5%)	3 (15.0%)			

*NS* non-significant, *S* significant, *HTN* hypertension, *MI* myocardial infraction, *CAD* coronary artery disease, *PCI* percutaneous coronary intervention, *CABG* coronary artery bypass graft

^a^Chi-square test

^b^Independent *t* test

There was no significant difference between both groups regarding age, sex, DM, hyperlipidemia, HTN, history of MI, family history of premature CAD, and previous PCI or CABG (*p* > 0.05).

#### Clinical presentation on admission (Table 2)

Patient symptoms were assessed according to chest pain and dyspnea. There was no significant difference between both groups regarding symptoms (*p* >0.05).

**Table 2:** Clinical presentation on admission

Symptoms and signs		EES group	BVS group	Test	***p*** value	Sig.
		No. = 40	No. = 20			
Chest pain	Negative	2 (5%)	0 (0%)	1.034	0.309	NS
	Positive	38 (95%)	20 (100%)			
Dyspnea	Negative	29 (72.5%)	17 (85%)	1.165	0.281	NS
	Positive	11 (27.5%)	3 (15%)			
BP	Normal	40 (100%)	20 (100%)	–	–	–
SBP	Mean ± SD	112.98 ± 3.5	114.67 ± 3.22	1.809	0.076	NS
	Range	100–120	100–120			
DBP	Mean ± SD	74.52 ± 1.7	74.80 ± 1.34	0.643	0.523	NS
	Range	70–80	70–80			
Pulse	Mean ± SD	74.05 ± 5.81	72.45 ± 4.97	1.053^b^	0.297	NS
	Range	60–86	65–76			
Murmur	No	29 (72.5%)	16 (80.0%)	0.400^a^	0.527	NS
	MR	11 (27.5%)	4 (20.0%)			

*BP* blood pressure, *SBP* systolic blood pressure, *DBP* diastolic blood pressure, *MR* mitral regurgitation, *NS* non-significant, *S* significant

^a^Chi-square test

^b^Independent *t* test

Regarding clinical examination, we found no significant difference between both group in respect to BP, pulse, and murmur (*p* > 0.05).

#### Laboratory investigations on admission (Table 3)

There was no significant difference between both groups as regard to CBC and serum creatinine (*p* > 0.05).

**Table 3:** Lab, echocardiographic data, and myocardial perfusion index (MPI) on admission

		EES group	BVS group	Test	***p*** value	Sig.
		No. = 40	No. = 20			
**Lab**						
Hemoglobin	Mean ± SD	13.5 ± 0.68	13.2 ± 0.89	1.448^b^	0.153	NS
	Range	12–14	12–14			
Creatinine	Mean ± SD	0.97 ± 0.13	0.99–0.08	− 0.639^b^	0.525	NS
	Range	0.4 – 1.2	0.8–1.2			
ECG	NAD	17 (42.5%)	7 (35.0%)	7.265^a^	0.202	NS
	Poor R wave progression	3 (7.5%)	2 (10.0%)			
	Q wave and inf.	7 (17.5%)	4 (20.0%)			
	Q wave and ant.	8 (20.0%)	3 (15.0%)			
	T inversion ant.	5 (12.5%)	1 (5.0%)			
	LAHB	0 (0.0%)	3 (15.0%)			
	NAD	17 (42.5%)	7 (35.0%)			
**Echo**						
EF (%)	Mean ± SD	57.55 ± 7.55	56.45 ± 11.11	0.453^b^	0.652	NS
	Range	45–73	40–85			
DD (grade)	I	1 (2.5%)	0 (0.0%)	0.508^a^	0.476	NS
	II	39 (97.5%)	20 (100.0%)			
Wall motion	No	23 (57.5%)	10 (50.0%)	2.161^a^	0.540	NS
	Anterior	13 (32.5%)	7 (35.0%)			
	Inferior	4 (10.0%)	2 (10.0%)			
	Lateral	0 (0.0%)	1 (5.0%)			
**MPI**						
Viable		13 (32.5%)	4 (20%)	2.063	0.840	NS
Ischemia anterior		5 (12.5%)	4 (20%)			
Ischemia inferior		6 (15%)	5 (25%)			
Ischemia lateral		2 (5%)	1 (5%)			
Mixed scar anterior		7 (17.5%)	3 (15%)			
Mixed scar inferior		7 (17.5%)	3 (15%)			

^a^Chi-square test

^b^Independent *t* test, *NS* non-significant, *S* significant, *CBC* complete blood count, *DD* diastolic dysfunction, *ECG* electrogardiogram

No significant difference was found between 2 groups as regard to ECG which reveal normal in 42% in EES group and 35% in BVS group (*p* = 0.202).

#### Echocardiography data on admission (Table 3)

No significant difference was found between both groups regarding EF (*p* = 0.652).

Diastolic dysfunction is present in 97% in EES group and 100% in BVS group (*p* = 0.476).

As regards to wall motion, there was no wall motion abnormalities in 57.5% in EES group and in 50% in BVS group, anterior hypokinesia 32.5% in EES group, and 35% in BVS group and inferior hypokinesia 10% in both groups and 5% lateral wall hypokinesia in BVS group.

#### Myocardial perfusion index on admission (MPI) (Table 3)

There was no significant difference between both groups regarding myocardial perfusion index which revealed viable myocardium in 32.5% in EES group and 20% in BVS group (*p* = 0.84).

### Angiographic and procedure characteristics

#### Intraprocedural and post-procedural aspect during intervention (Table 4 and Fig. 2)

Baseline lesion data presented in Table 4 revealed LAD and RCA were the most frequent index lesion in total study population and each presents 45% of total study population, while LCX presents 10%.

**Table 4:** Intraprocedural and post-procedural aspect during intervention

PCI		DES group	Absorb group	Test	***p*** value	Sig.
		No. = 40	No. = 20			
Artery	LAD	18 (45.0%)	9 (45.0%)	0.000^a^	1.000	NS
	RCA	18 (45.0%)	9 (45.0%)			
	LCX	4 (10.0%)	2 (10.0%)			
Antegrade or retrograde	Antegrade	30 (75.0%)	15 (75.0%)	0.000^a^	1.000	NS
	Retrograde	10 (25.0%)	5 (25.0%)			
J score	Median (IQR)	3 (3 – 4)	3 (3–4)	− 0.679^c^	0.497	NS
	Range	3–4	2–4			
Pre-dilatation	Done	40 (100.0%)	20 (100.0%)	–	–	–
Post-dilatation	Not done	2 (5.0%)	0 (0.0%)	1.034^a^	0.309	NS
	Done	38 (95.0%)	20 (100.0%)			
Stent length	Mean ± SD	32.53 ± 9.46	29.40 ± 8.61	1.242^b^	0.219	NS
	Range	15–48	18–38			
DAPT	Given	40 (100.0%)	20 (100.0%)	–	–	–
TIMI flow intraprocedure	II	2 (5.0%)	0 (0.0%)	1.034^a^	0.309	NS
	III	38 (95.0%)	20 (100.0%)			
Coronary perforation	occurred	0 (0%)	1 (0.05%)	0.000^a^	1.000	NS

*NS* non-significant, *S* significant

^a^Chi-square test

^b^Independent *t* test

^c^Mann-Whitney test

**Fig. 2:**
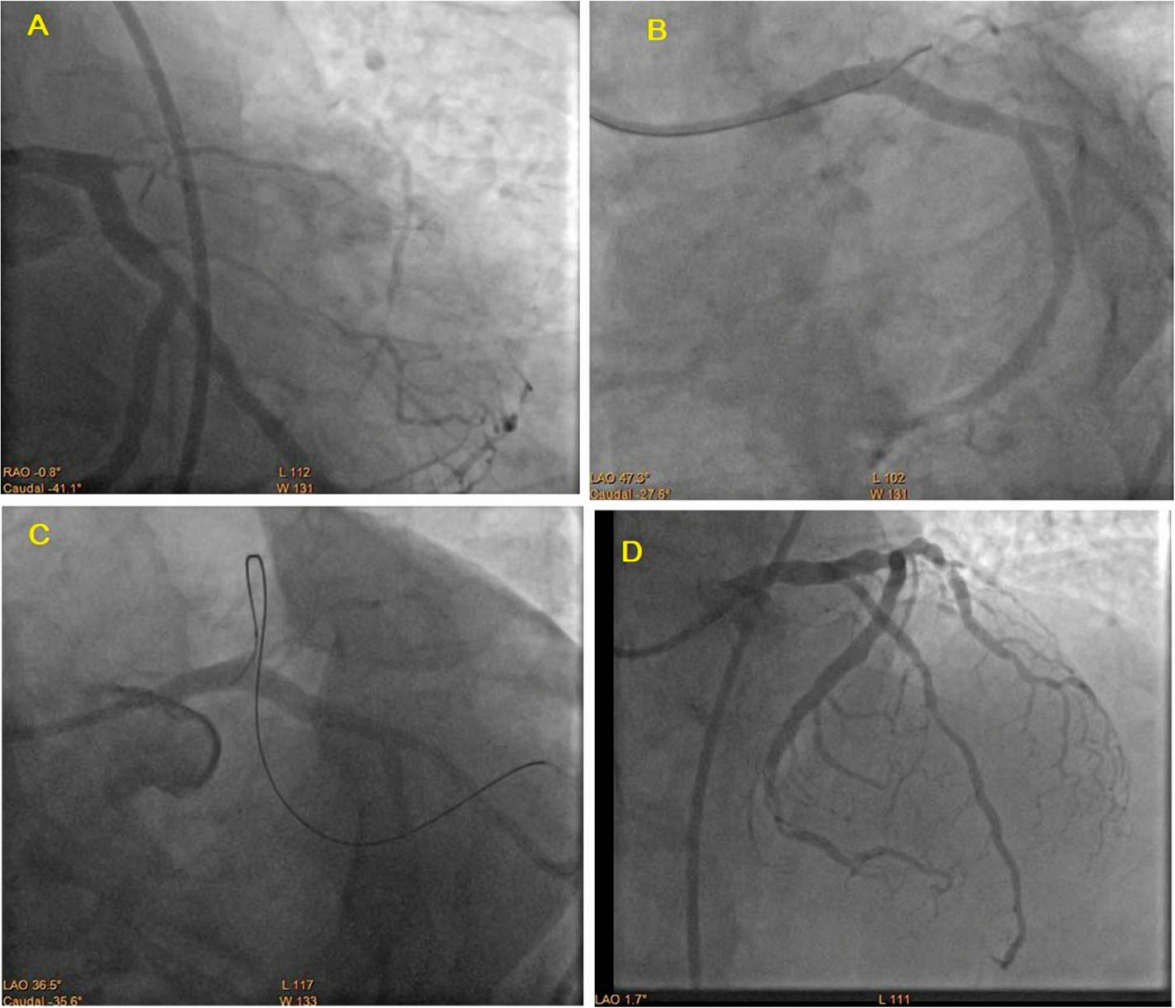
**a** Coronary angiography of CTO of osteal LAD with J-CTO score complexity = 2. **b** Trial of antegrade approach **c** Successful retrograde approach. **d** Successful revascularization of total LAD by DES

The procedure was done most frequently by antegrade approach in 75% in both groups and by retrograde approach in 25% in both groups. Retrograde approach was failed to cross CTO site in another 5 (4.0%) patients; those 5 patients had another trial of successful antegrade approach. There were no significant differences in respect to angiographic characteristics of the lesion between success and failure groups.

Evaluation of index lesion was done by J score which revealed no significant difference between both groups (*p* = 0.022) in J score.

Post-dilatation was done in 95% of DES group and 100% of BVS group with no significant different between both groups (*p* = 0.309).

No significant difference was found in stent size between both groups (*p* = 0.219).

Coronary perforation occurred only in one patient (0.05%) of BVS group and was managed successfully by simple balloon inflation without progressing to cardiac tamponade.

Concerning in-hospital outcome in the current study, there was no reported mortality during hospitalization period, *Q* wave myocardial infarction developed in 1.6% of study population and non *Q* wave myocardial infarction developed in 2.4%.

### Clinical outcome and follow-up

#### All MACE results

##### Post-procedural follow-up (Table 5)

All study population of both groups was on dual anti-platelet for at least 1 year post-procedural. TIMI III flow was achieved in 95% in DES group and 100% of BVS group with reveal no significant difference (*p* = 0.309).

**Table 5:** Post-procedural follo-up

		EES group	BVS group	*χ2* test	***p*** value	Sig.
		No. = 40	No. = 20			
**Mortality**						
1 month	Negative	40 (100%)	20 (100%)	–	–	–
	Positive	0 (0%)	0 (0%)			
6 months	Negative	40 (100%)	20 (100%)	–	–	–
	Positive	0 (0%)	0 (0%)			
1 year	Negative	40 (100%)	20 (100%)	–	–	–
	Positive	0 (0%)	0 (0%)			
**Non-fatal MI**						
1 month	Negative	40 (100%)	20 (100%)	–	–	–
	Positive	0 (0%)	0 (0%)			
6 months	Negative	40 (100%)	20 (100%)	–	–	–
	Positive	0 (0%)	0 (0%)			
1 year	Negative	39 (97.5%)	20 (100%)	0.508	0.476	NS
	Positive	1 (2.5%)	0 (0%)			
**UA requiring hospitalization**						
1 month	Negative	40 (100%)	20 (100%)	–	–	–
	Positive	0 (0%)	0 (0%)			
6 months	Negative	25 (62.5%)	14 (70%)	0.330	0.566	NS
	Positive	15 (37.5%)	6 (30.0%)			
1 year	Negative	39 (97.5%)	20 (100%)	0.508	0.476	NS
	Positive	1 (2.5%)	0 (0%)			
**Multi-slice CT (MSCT)**						
6 months	Patent	36 (90%)	18 (90%)	0.000	1.000	NS
	Occluded	4 (10%)	2 (10%)			
1 year	Patent	36 (90%)	18 (90%)	0.000	1.000	NS
	Occluded	4 (10%)	2 (10%)			

*MI* myocardial infarction, *UA* unstable angina

##### Mortality during the follow-up period (Table 5)

Follow-up of all study population up to 1 year revealed no mortality reported in any group.

##### Non-fatal MI (Table 5)

During the follow-up period, there was no reported MI in any group at the 1st and 6th month of follow up. However, there was one MI case in EES group at 1 year follow up while none in BVS group (*p* = 0.476).

##### Unstable angina (UA) requiring hospitalization (Table 5)

There was no significant difference between both groups as regard unstable angina requiring hospitalization at 6 months and 1 year follow-up (*p* = 0.566 and 0.476 respectively).

#### The CT Coronary angiography results

##### Multi-slice CT coronary angiography (MSCT) (Table 5 and Fig. 3)

No significant difference was found between both groups at 6 months follow-up by MSCT which revealed patent stent in 90% and 10% occluded stent in both groups (*p* = 1.000).

**Fig. 3:**
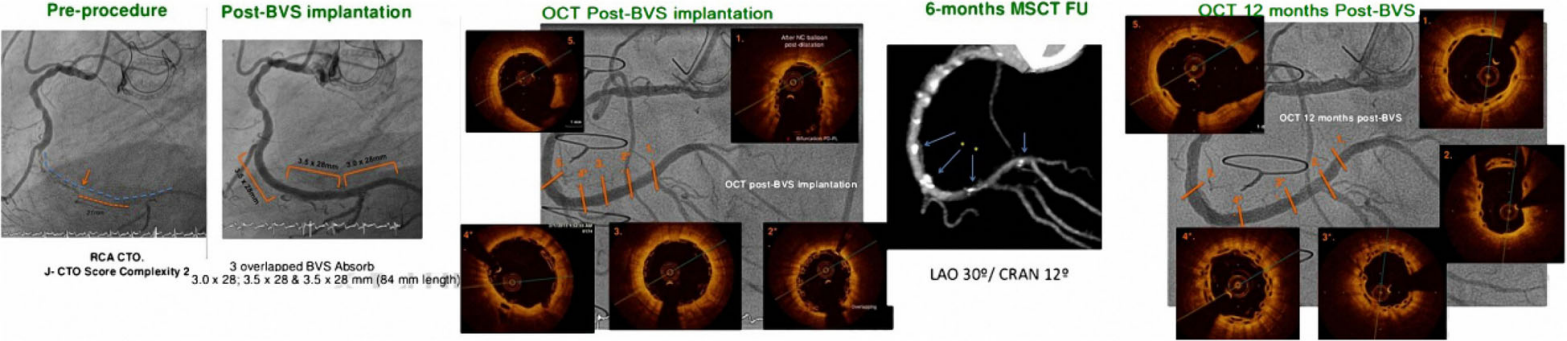
First panel from the Lt. side: Coronary angiography of CTO of mid RCA with J-CTO score complexity = 2. Second panel: Post-BVS implantation of 3 overlapped BVS Absorb (3.0 × 28; 3.5 × 28, and 3.5 × 28 mm (84 mm length). Third panel: OCT Post-BVS implantation. Fourth panel: 6 months MSCT follow-up. Fifth panel: OCT 12 months post-BVS

## Discussion

### Baseline clinical characteristics

Regarding the age of patients in the current study ranged from 34 to 80 years, with mean age of (58 ± 8.6) years. The mean age of the sample in the present study is comparable to the mean age of patients enrolled in study by Hoye et al. (2005) (59.8 ± 11.1 years); however, Morino et al. (2010) reported a higher mean age in his study on outcomes of PCI in patients with CTOs (66.9 ± 11 years) [20, 21].

The baseline characteristics and risks factors were comparable between the two study groups.

### Angiographic and procedure characteristics

Regarding angiographic procedure in current study, we found that occluded stent just occurred in 2 patients (10%) of BVS group and 4 patients (10%) of EES group at 6 months follow-up without significant difference between both groups. This large percent can be explained by our small sample size.

Our study in comparable with primary end point of absorb III trials which showed target lesion failure (TLF) at 12 months, this trial included 2008 patients (revascularization by BVS was 1322 and revascularization by DES 686) which showed TLF was 7.8% in BVS group and 6.1% in DES group. So absorb III trials provides adequate result of non-inferiority of BVS against DES in treatment of CTO lesions [22].

Absorb-CTO-registry which included 35 patients with true CTO lesion clearly showed that BVS use in CTO in feasible with good mid-term efficacy and 6 months follow-up by multi-slice computed tomography identifies only 2 cases scaffold re-occlusion without any major adverse event. Safety and feasibility of BVS implantation in CTO has been confirmed in this registry [23]. These results were matched with our study results.

Another Ghost-CTO-registry which included 21 patients who had CTO lesions which treated by BVS and followed up after 1 year by OCT showed overall favorable vascular response and healing profile [24]. These results are also comparable with our study.

In our study, the lesion location of CTOs were in left anterior descending artery (LAD) in 45% of cases, right coronary artery (RCA) in 45% of cases, and left circumflex artery (LCX) in 10% of cases.

In the current study, all patients had initial attempt of wiring through antegrade approach, if antegrade approach was failed, then retrograde approach was tried.

Retrograde approach has been described initially via the bypass grafts and more recently the use of septal collaterals has been described to be safe and efficient. Retrograde approach for recanalization of CTO has gained popularity recently with increased success rates as shown in several case reports and small series of selected patients [25].

Retrograde approach requires good special devices, such as micro catheters, dedicated guide wires, shorter guide catheters, or longer length guide wires, and should be performed with experienced operators and support staff.

Retrograde approach was successful in 15 (25%) patients, and was failed to cross CTO site in another 5 (4.0%) patients, those 5 patients had another trial of successful antegrade approach. There were no significant differences in respect to angiographic characteristics of the lesion between success and failure groups.

However, the conventional factors such as severe calcification, severe tortuosity, significant side branch at the CTO site and CTO length, which are shown to be independent predictors of successful procedure via antegrade approach in various studies [26] are not shown to have any significant impact on outcomes during retrograde recanalization in our study.

There was no difference between both groups possibly due to that conventional factors are not important when the retrograde techniques are applied, as the wire manipulation is done in retrograde fashion to gain access to either true or false lumen and to complete the procedure with adjunctive techniques.

Regarding procedure related complication in the current study, there was no reported cases of mortality or stent thrombosis, this might be due to operators experience and use of intense anti-platelet regimen and limited number of patients.

One of the most fatal complications during CTO intervention is coronary perforation and tamponade [21].

In spite of angiographic coronary perforation was frequently observed during CTO treatment, so evaluating the clinical significance of angiographic perforation is critically important, and goal must be to avoid cardiac tamponade.

In the current study, coronary perforation occurred only in one patient (0.05%) of BVS group and was managed successfully by simple balloon inflation without progressing to cardiac tamponade.

Similar figure was reported by Morino et al. (2010) and they reported incidence of 0.4%. These figures are considered low compared with the previous reports (a range of 0.8% to 1.9%) [21].

Potential hypotheses to explain low incidence of cardiac tamponade are adequate hemostatic procedures as needed, reduction of prolonged and aggressive antegrade guide wire manipulation. Thanks to the introduction of retrograde approaches. Although a careful evaluation is required, procedural continuation could be considered with angiographic coronary perforation.

### Clinical outcome and follow-up

Concerning in-hospital outcome in the current study, there was no reported mortality during hospitalization period, *Q* wave myocardial infarction developed in 1.6% of study population and non *Q* wave myocardial infarction developed in 2.4%.

Close figures were reported by Lemos et al. (2004) who reported no death, TLR, TVR or Q wave MI, but incidence of 7.4% of non Q wave MI [27].

After the introduction of DES, several studies demonstrated that DES markedly reduce the incidence of angiographic restenosis and repeat revascularization in selected patients with relatively noncomplex lesions. In patients with CTO lesions, there are several trials comparing the efficacy of DES with BMS but studies comparing individual DESs against BVS are limited [28].

Regarding 12-month clinical follow-up in our study, no mortality occurred in all study population in both arms, and at 6 months follow-up unstable angina occurred in 37% with EES and 30% in BVS stent group which agreement with other previous studies from multiple randomized controlled trials (RCT) while revealing no significance between 2 groups about MACE at 12 months follow-up.

Our study shows non inferiority of BVS stent in CTO comparable with EES which matched with absorb III trial. However, our study of small population size limit data to show advantage of BVS on DES.

The knowledge that PCI does not prevent MI or prolong life in stable or stabilized coronary disease is well established. This was reinforced by the COURAGE and OAT trials in which PCI did not provide incremental benefits in hard end points such as MI or mortality above contemporary medical management. However, because COURAGE did not address CTOs specifically and OAT addressed only subacute post-MI occlusions, the generalized ability of these trials to CTO is not clear [29]

Furthermore, the COURAGE nuclear substudy demonstrated event reduction in patient with moderate to severe ischemia. The CTO populations are often enriched with patients with high ischemic burden who may likely benefit from recanalization [30].

Coronary BVS are attractive therapeutic option in interventional cardiology; BVS backbone is made of poly-lactoide and coated by thin layer of poly-dilactrole. It releases everolimus and is fully degraded to H_2_O and CO_2_ in 2–3 years. BVS seems to offer several theoretical advantage over metallic stent, as it gives temporary mechanical support of vessel wall; therefore, long-term endothelial function and structure are not affected, and a possible future surgical revascularization is not compromised [31].

### Limitations of the study

This study was limited by its small sample size in patients from a two medical centers. Second generation BVS was not used for comparison as it is still under development. We need longer follow up to detect the outcome of BVS.

#### Clinical implication

- ○ We believe that 1st generation everolimus-eluting bioresorbable vascular scaffold (BVS) stent associated with less complication and less restenosis rate than everolimus-eluting stent (EES) in chronic total occlusion (CTO) recanalization guided by intracoronary imaging.
- ○ Further follow-up data should be done on a large scale in order to provide evidence for how to follow-up and treat those patients.

## Conclusion

In light of our findings, we conclude that our study showed that 1st generation everolimus-eluting BVS is non-inferior to EES for CTO revascularization. Further studies are needed to clearly state which new smaller footprint BVS, faster reabsorption, magnesium-based less thrombogenicity, and advanced mechanical properties is under development. We cannot dismiss the efficacy and safety of new BVS technology. BVS by Abbott should focus on simple and short lesion. Various studies did not provide better outcome with long-term patency in tortuous, long, calcified lesions. Biotronik, biomime bioabsorbable stents came in and needs more comparative study.

## Data Availability

Our prospective comparative cross-sectional study data used to support the findings of this study are available from the corresponding author upon request.

## Abbreviations

2h-PPBG: 2-h post-prandial blood sugar
ABPM: Ambulatory blood pressure
AF: Atrial fibrillation
BVS: Bioresorbable vascular scaffold
CABG: Coronary artery bypass graft
CAD: Coronary artery disease
CRP: C-reactive protein
CTO: Chronic total occlusion
DES: Drug-eluting stents
ECG: Electrocardiogram
EDV: End-diastolic volume
EES: Everolimus-eluting stent
EF: LV ejection fraction
ESV: End-systolic volume
FBS: Fasting blood sugar
HBPM: Home blood pressure monitoring
HDL: High-density lipoprotein
IHD: Ischemic heart disease
LBBB: Left bundle branch block
LDL: Low-density lipoprotein
MACE: Major adverse cardiovascular events
MPI: Myocardial perfusion index
MSCT: Multi-slice CT coronary angiography
NHIS: National Health Interview Survey
OCT: Optical coherence tomography
OGTT: Oral glucose tolerance test
PCI: Percutaneous coronary intervention
QCA: Quantitative coronary angiography
RBBB: Right bundle branch block
TG: Triglycerides
TTE: Transthoracic echocardiography
TVR: Target vessel revascularization

## References

[1] Giordano A, Ferraro P, Corcione N, Messina S, Maresca G, Coscioni E, Biondi-Zoccai G (2016). Successful treatment of recurrent carotid in-stent restenosis and drug-eluting balloon failure with a coronary bioresorbable vascular scaffold: a case report. Int J Surg Case Rep.

[2] Chan W, Shah A, Džavík V, Overgaard CB (2014). Complex coronary artery bifurcation treatment utilizing everolimus-eluting bioresorbable vascular scaffolds and optical coherence tomography. Coron Artery Dis.

[3] Dalos D, Gangl C, Roth C, Krenn L, Scherzer S, Vertesich M, Lang I, Maurer G, Neunteufl T, Berger R, Delle-Karth G (2016). Mechanical properties of the everolimus-eluting bioresorbable vascular scaffold compared to the metallic everolimus-eluting stent. BMC cardiovasc dis.

[4] Dudek D, Onuma Y, Ormiston JA, Thuesen L, Miquel-Hebert K, Serruys PW (2012). Four-year clinical follow-up of the ABSORB everolimus-eluting bioresorbable vascular scaffold in patients with de novo coronary artery disease: the ABSORB trial. EuroIntervention.

[5] Shehata IE, Cheng CI, Sung PH, Ammar AS, El-Sherbiny IA, Ghanem IG (2018). Predictors of myocardial functional recovery following successful reperfusion of acute ST elevation myocardial infarction. Echocardiography.

[6] Nef H, Wiebe J, Achenbach S, Münzel T, Naber C, Richardt G, Mehilli J, Wöhrle J, Neumann T, Biermann J, Zahn R (2016). Evaluation of the short-and long-term safety and therapy outcomes of the everolimus-eluting bioresorbable vascular scaffold system in patients with coronary artery stenosis: Rationale and design of the German–Austrian ABSORB RegIstRy (GABI-R). Cardiovasc Revasc.

[7] Ielasi A, Tespili M (2014). Current and future perspectives on drug-eluting bioresorbable coronary scaffolds. Futur Cardiol.

[8] Kimura T, Kozuma K, Tanabe K, Nakamura S, Yamane M, Muramatsu T, Saito S, Yajima J, Hagiwara N, Mitsudo K, Popma JJ (2015). A randomized trial evaluating everolimus-eluting Absorb bioresorbable scaffolds vs. everolimus-eluting metallic stents in patients with coronary artery disease: ABSORB Japan. Eur Heart J.

[9] Abizaid A, Costa RA, Schofer J, Ormiston J, Maeng M, Witzenbichler B, Botelho RV, Costa JR, Chamié D, Abizaid AS, Castro JP (2016). Serial multimodality imaging and 2-year clinical outcomes of the novel DESolve novolimus-eluting bioresorbable coronary scaffold system for the treatment of single de novo coronary lesions. JACC Cardiovasc Interv.

[10] Brugaletta S, Gomez-Lara J, Garcia-Garcia HM, Heo JH, Farooq V, van Geuns RJ, Chevalier B, Windecker S, McClean D, Thuesen L, Whitbourn R (2012). Analysis of 1 year virtual histology changes in coronary plaque located behind the struts of the everolimus eluting bioresorbable vascular scaffold. Int J Cardiovasc imaging.

[11] Ellis SG, Kereiakes DJ, Metzger DC, Caputo RP, Rizik DG, Teirstein PS, Litt MR, Kini A, Kabour A, Marx SO, Popma JJ (2015). Everolimus-eluting bioresorbable scaffolds for coronary artery disease. N Engl J Med.

[12] Huang LY, Yang MC, Tsou HM, Liu TY (2019). Hemocompatibility and anti-fouling behavior of multilayer biopolymers immobilized on gold-thiolized drug-eluting cardiovascular stents. Colloids and Surfaces B: Biointerfaces.

[13] Williams B, Mancia G, Spiering W, Agabiti Rosei E, Azizi M, Burnier M, Clement DL, Coca A, De Simone G, Dominiczak A, Kahan T (2018). 2018 ESC/ESH Guidelines for the management of arterial hypertension: The Task Force for the management of arterial hypertension of the European Society of Cardiology (ESC) and the European Society of Hypertension (ESH). Eur Heart J.

[14] Chamberlain JJ, Rhinehart AS, Shaefer CF, Neuman A (2016). Diagnosis and management of diabetes: synopsis of the 2016 American Diabetes Association Standards of Medical Care in Diabetes. Ann Intern Med.

[15] Grundy SM, Stone NJ (2019). 2018 American Heart Association/American College of Cardiology/Multisociety Guideline on the Management of Blood Cholesterol–Secondary Prevention. JAMA Cardiol.

[16] Barua RS, Rigotti NA, Benowitz NL, Cummings KM, Jazayeri MA, Morris PB, Ratchford EV, Sarna L, Stecker EC, Wiggins BS (2018). ACC expert consensus decision pathway on tobacco cessation treatment: a report of the American College of Cardiology Task Force on Clinical Expert Consensus Documents. J Am Coll Cardiol.

[17] Bachmann JM, Willis BL, Ayers CR, Khera A, Berry JD (2012). Association between family history and coronary heart disease death across long-term follow-up in men: the Cooper Center Longitudinal Study. Circulation.

[18] Lang RM, Badano LP, Mor-Avi V, Afilalo J, Armstrong A, Ernande L, Flachskampf FA, Foster E, Goldstein SA, Kuznetsova T, Lancellotti P (2015). Recommendations for cardiac chamber quantification by echocardiography in adults: an update from the American Society of Echocardiography and the European Association of Cardiovascular Imaging. J Am Soc Echocardigr.

[19] Christopoulos G, Karmpaliotis D, Alaswad K, Yeh RW, Jaffer FA, Wyman RM, Lombardi WL, Menon RV, Grantham JA, Kandzari DE, Lembo N (2015). Application and outcomes of a hybrid approach to chronic total occlusion percutaneous coronary intervention in a contemporary multicenter US registry. Int J Cardiol.

[20] Hoye A, van Domburg RT, Sonnenschein K, Serruys PW (2005). Percutaneous coronary intervention for chronic total occlusions: the Thoraxcenter experience 1992–2002. Eur Heart J.

[21] Morino Y, Kimura T, Hayashi Y, Muramatsu T, Ochiai M, Noguchi Y, Kato K, Shibata Y, Hiasa Y, Doi O, Yamashita T (2010). In-hospital outcomes of contemporary percutaneous coronary intervention in patients with chronic total occlusion: insights from the J-CTO Registry (Multicenter CTO Registry in Japan). JACC Cardiovasc Interv.

[22] Stone GW, Kimura T, Gao R, Kereiakes DJ, Ellis SG, Onuma Y, Chevalier B, Simonton C, Dressler O, Crowley A, Ali ZA (2019). Time-varying outcomes with the absorb bioresorbable vascular scaffold during 5-year follow-up: a systematic meta-analysis and individual patient data pooled study. JAMA Cardiol.

[23] Capodanno D, Gori T, Nef H, Latib A, Mehilli J, Lesiak M, Caramanno G, Naber C, Di Mario C, Colombo A, Capranzano P (2015). Percutaneous coronary intervention with everolimus-eluting bioresorbable vascular scaffolds in routine clinical practice: early and midterm outcomes from the European multicentre GHOST-EU registry. EuroIntervention.

[24] La Manna A, Miccichè E, D'Agosta G, Pereira GT, Attizzani GF, Capranzano P, Capodanno D, Tamburino C (2018). Vascular response and healing profile of everolimus-eluting bioresorbable vascular scaffolds for percutaneous treatment of chronic total coronary occlusions: a one-year optical coherence tomography analysis from the GHOST-CTO registry. Inter J Cardiol.

[25] Sheiban I, Moretti C, Omedé P, Sciuto F, Bollati M, Laudito A, Trevi GP, Biondi-zoccai GG (2007). The retrograde coronary approach for chronic total occlusions: Mid-term results and technical tips & tricks. J Interv Cardiol.

[26] Noguchi T, Miyazaki MDS, Morii I, Daikoku S, Goto Y, Nonogi H (2000). Percutaneous transluminal coronary angioplasty of chronic total occlusions. Determinants of primary success and long-term clinical outcome. Catheter Cardiovasc Interv.

[27] Lemos PA, Serruys PW, van Domburg RT, Saia F, Arampatzis CA, Hoye A, Degertekin M, Tanabe K, Daemen J, Liu TK, McFadden E (2004). Unrestricted Utilization of Sirolimus-Eluting Stents Compared With Conventional Bare Stent Implantation in the “Real World” The R apamycin-E luting S tent E valuated A t R otterdam C ardiology H ospital (RESEARCH) Registry. Circulation.

[28] Nakamura S, Muthusamy TS, Bae JH, Cahyadi YH, Udayachalerm W, Tresukosol D (2005). Impact of sirolimus-eluting stent on the outcome of patients with chronic total occlusions. Am J Cardiol.

[29] Shaw LJ, Berman DS, Maron DJ, Mancini GB, Hayes SW, Hartigan PM, Weintraub WS, O’Rourke RA, Dada M, Spertus JA, Chaitman BR (2008). Optimal medical therapy with or without percutaneous coronary intervention to reduce ischemic burden. Circulation.

[30] Sirnes PA, Golf S, Myreng Y, Mølstad P, Albertsson P, Mangschau A, Endresen K, Kjekshus J (1998). Sustained benefit of stenting chronic coronary occlusion: long-term clinical follow-up of the Stenting in Chronic Coronary Occlusion (SICCO) study. J Am Coll Cardiol.

[31] Ni L, Chen H, Luo Z, Yu Y (2020). Bioresorbable vascular stents and drug-eluting stents in treatment of coronary heart disease: a meta-analysis. J Cardiothorac Surg.

